# Experimentally testing and assessing the predictive power of species assembly rules for tropical canopy ants

**DOI:** 10.1111/ele.12403

**Published:** 2015-01-27

**Authors:** Tom M Fayle, Paul Eggleton, Andrea Manica, Kalsum M Yusah, William A Foster

**Affiliations:** 1 Institute of Entomology, Biology Centre of Academy of Sciences Czech Republic and Faculty of Science, University of South BohemiaBranišovská 31, 370 05, České Budějovice, Czech Republic; 2 Forest Ecology and Conservation Group, Imperial College London, Silwood Park CampusBuckhurst Road, Berkshire, SL5 7PY, UK; 3 Institute for Tropical Biology and Conservation, Universiti Malaysia Sabah88400, Kota Kinabalu, Sabah, Malaysia; 4 Life Sciences Department, Natural History MuseumCromwell Road, London, SW6 5BD, UK; 5 Evolutionary Ecology Group, Department of Zoology, University of CambridgeCambridge, CB2 3EJ, UK; 6 Insect Ecology Group, University Museum of Zoology CambridgeDowning Street, Cambridge, CB2 3EJ, UK

**Keywords:** *Asplenium*, bird's nest fern, diffuse competition, epiphyte, Formicidae, microcosm, mutualism, nearest neighbour competition, rainforest

## Abstract

Understanding how species assemble into communities is a key goal in ecology. However, assembly rules are rarely tested experimentally, and their ability to shape real communities is poorly known. We surveyed a diverse community of epiphyte-dwelling ants and found that similar-sized species co-occurred less often than expected. Laboratory experiments demonstrated that invasion was discouraged by the presence of similarly sized resident species. The size difference for which invasion was less likely was the same as that for which wild species exhibited reduced co-occurrence. Finally we explored whether our experimentally derived assembly rules could simulate realistic communities. Communities simulated using size-based species assembly exhibited diversities closer to wild communities than those simulated using size-independent assembly, with results being sensitive to the combination of rules employed. Hence, species segregation in the wild can be driven by competitive species assembly, and this process is sufficient to generate observed species abundance distributions for tropical epiphyte-dwelling ants.

## Introduction

Communities can be thought as the culmination of a sequence of serially dependent colonisation events, where the success of each invasion depends on a set of assembly rules (Weiher & Keddy 2001). For example, the invasion success of a given species might be affected by local environmental conditions (Leibold *et al*. 2010), or by the presence of one or more other species (Kunstler *et al*. 2012). Understanding whether these assembly rules exist, how they operate, and their effects on overall community structure has been a central theme in community ecology (Diamond 1975; Connor & Simberloff 1979; Gotelli 2000).

Competition has been argued to be a major determinant of natural species assemblies (e.g. Kunstler *et al*. 2012). A specific prediction from assembly rules based on competition among species with similar niches is that these species should be less likely to co-occur when compared with species with dissimilar niches (Kraft *et al*. 2008). Co-occurrence can be assessed using null models, which test the observed pattern of co-occurrence across multiple sampling locations against the expected levels when species are randomly assembled under constraints preserving particular aspects of the observed communities (Gotelli 2000). However, significant species segregation across sampling locations is not sufficient to conclude that competitive assembly rules are driving this pattern. This is because other processes, such as environmental filtering (Leibold *et al*. 2010), and dispersal limitation (Hubbell 2001) can also give rise to species segregation. Ironically, this issue is compounded by the increasingly large observational datasets that are becoming available for analysis, in which even very weak interactions can be detected. Hence, it is necessary to conduct experimental manipulations to determine: (1) whether segregation relates to competitive species assembly rather than some alternative process and (2) if species do compete, the strength of this interaction. Such manipulations have detected segregation within functional groups, and have been predominantly conducted on plant communities (Price & Pärtel 2013) (although see Ehmann & MacMahon 1996; Fincke 1999). Since such experiments are only rarely performed, the prevalence of competitive species assembly remains unknown.

A further challenge facing species assembly research is that proving a particular assembly rule operates under a given experimental setting does not necessarily imply that it strongly affects community structure in the wild, since other unquantified drivers may be more important (see Kikvidze *et al*. 2011 for a discussion of this general problem for ecology). For example, a particular pair of species might compete very strongly in the laboratory, but rarely interact in the wild. This could happen if both species are uncommon, or if they use different parts of a habitat. Hence, experimental tests of species assembly rules that do not account for community-level context may fail to reflect the importance of competitive species assembly.

One strategy to overcome this problem, and a natural progression beyond comparing observed communities to simulated ones that assume no species interactions (null modelling), is to simulate community assembly using the proposed rule with parameters derived using relevant (independent) experiments. If the resulting simulated communities derived using this process-based approach resemble the observed ones then this provides strong evidence that the proposed assembly rule is important (Grimm *et al*. 2005). Furthermore, if simulations incorporating the same constraints (e.g. community size, meta-community and species richness), but using a null assembly rule instead, fail to recreate observed community patterns then this argues for the importance of the focal rule in structuring real communities. Most importantly, the use of experimentally derived parameter values to quantify the strength of species interactions provides an independent test of the role of a particular rule, thus avoiding the potential circularity of testing the importance of a rule using only parameters from the original, observational dataset.

Here, we test for the existence of competitive species assembly using the ant communities inhabiting epiphytic bird's nest ferns (*Asplenium* spp.) in tropical rain forest. *Asplenium* ferns derive their nutrients from intercepted leaf litter (Turner *et al*. 2007) and the resulting mass of decomposing litter supports diverse arthropod communities, with ants being most abundant (Turner & Foster 2009). In contrast to the many ant–epiphyte interactions in which a single mutualistic species of ant dominates, multiple colonies of different species of ant coexist, with no association between ant and fern species (Fayle *et al*. 2012). This is because the ferns are unable to reward individual cooperating ant colonies selectively since the ants inhabit the core of fern roots and decomposing leaf litter, rather than discrete structures adapted for ant inhabitation (Fayle *et al*. 2012). Consequently, in large ferns colonies of up to 12 species of ant can coexist in close proximity, making them ideal arenas in which to test assembly rule models (Srivastava *et al*. 2004). We focus on testing whether ants with similar body sizes are more likely to experience competition during species assembly (Davidson 1985), and whether these size-based rules could drive ant community structure. Species with similar body sizes are expected to co-occur rarely, since they are likely to utilise the same resources (Dayan & Simberloff 2005). Although this hypothesis has been tested before for ants, the results have been equivocal, with patterns of weak segregation, random co-occurrence, and aggregation (Foitzik & Heinze 1999; Gotelli & Ellison 2002; King 2007; Donoso 2014). Specifically we test how well different relationships between trait (i.e. body size) similarity and strength of competition predict community structure, and whether only the most ecologically similar resident species compete, or if all resident species determine whether a new invading species is successful.

We demonstrate that species in wild canopy ant communities in Malaysian Borneo are segregated on the basis of body size. Furthermore, the success of experimentally introduced ant colonies depends on the relative body sizes of the resident and introduced species, with similar-sized species experiencing reduced colonisation probability. The range of body size differences over which competition operates in our experiments matches the range over which there is reduced co-occurrence between species in wild communities. Finally, we show that communities simulated using a body-size based assembly rule, parameterised with the experimental results, display similar diversity to wild communities, while communities simulated under the assumption that assembly is size-independent do not display this high diversity.

## Material and Methods

### Field site and ant collections

We studied the ant communities inhabiting bird's nest ferns (*Asplenium* spp.) from a homogeneous area of lowland primary dipterocarp rain forest in Danum Valley Conservation Area, Sabah, Malaysia (117°49′ E, 5°01′ N). Ferns were collected using a combination of rope access techniques and ladders and were dissected using a saw and secateurs (see Appendix S1 for further details). Ant body size was measured as Weber's length, the maximum length of the alitrunk, which is usually the longest unarticulated ant body segment (Sanders *et al*. 2007).

### Observations of ant co-occurrence patterns in wild ferns

To assess co-occurrence in wild communities, ant assemblages were collected from the cores of 86 epiphytic ferns randomly selected from those accessible in twenty 20 × 100 m transects, which were randomly placed within 2 km of the field centre (see Fayle *et al*. 2012). A species by samples matrix was constructed showing presences and absences of all ant species in all ferns. Since we could not be certain of detecting multiple colonies of the same species (due to polygyny and potentially missed queens), all analyses described here focus exclusively on the effects of interspecific competition based on presence/absence data.

We quantified species segregation based on body size (as expected from niche-based assembly rules) by plotting the cumulative number of co-occurrences between species pairs as a function of increasing difference in body size (log_10_ transformed). We then tested the significance of this relationship by generating 10 000 null matrices of ant presence/absence, with occurrences across species and species richness across sites constrained to the observed values [fixed row and column totals, quasi-swap algorithm, *commsimulator* function in R package *vegan* (Oksanen *et al*. 2008)]. For each null matrix, we estimated the relationship between the cumulative number of co-occurrences and body-size difference, thus providing us with confidence intervals and significance values for the observed numbers of co-occurrences under the null hypotheses of random species co-occurrence (Appendix S2). This method is derived from Ripley's K-function, which assesses spatial patterns (Ripley 1976), but our method uses differences between species in the trait of interest (i.e. size) rather than spatial distance. We also quantified statistical power, since this is expected to be low for smaller size differences, where there are few pairs of co-occurring species (Appendix S1). Furthermore, we tested whether any segregation between similar-sized species correlated with the sizes of those species, and also whether size-based co-occurrence might be explained by phylogenetic relatedness (Appendix S1, Figs S2 and S3).

### Invasion of experimental fern-ant communities

We then conducted experiments to investigate whether any patterns of species segregation in wild communities were driven by competition. Specifically we used laboratory-based invasions to test whether the presence of similar-sized ant species prevented invasion of an incoming colony. An undescribed species of *Diacamma*, often found in the ferns (*Diacamma* #200, see Fayle *et al*. 2012), was used as the invader. We created three invasion scenarios: empty ferns (control, *n* = 50), ferns inoculated with one out of eight possible resident species, including the invader (*n* = 14 per species, see Table 1), and ferns with three different residents (*n* = 4 for each of 15 combinations of six resident species). For treatments with multiple residents, two species used in the single species treatment were not used (*Diacamma* #200, the invader, and a species of *Leptogenys*, which was the least common of the other species). These species were excluded in order to reduce the number of combinations of different ant species and therefore allow replication of each combination. Resident species were left for 48 h to acclimate before invasion. All colonies had queens present, since this might affect aggression levels. Invading *Diacamma* #200 colonies consisted of six workers and one gamergate reproductive, a colony comparable in size to those found in wild ferns of the same size. In each replicate, an invading colony was introduced into a fern, which was supported on a fluon-coated cylinder above a fluon-coated container, and left for 24 h with ants ejected from the fern falling into the container. Competition manifested as direct attacks between workers of different colonies, with ants sometimes being thrown from the edge of the fern (Appendix S3). We considered two measures of invasion success: (1) reproductive invasion success (a binary response), defined as the gamergate (reproductive) entering the fern; and (2) the proportion of the invading colony successfully entering the fern.

**Table 1:** The identity of the resident ant species affected experimental invasion probability by *Diacamma* #200 for ferns inhabited by a single ant species compared with empty control ferns. Two measures of invasion success were used: proportion of trials in which the incoming reproductive remained at the end of the trial, and mean proportion of workers remaining at the end of the trial (regardless of whether the reproductive remained). Difference in logged body size between the invader and the defender is also presented

Resident species	Body size (Weber's length, mm)	Difference in log_10_ body size	Trials	Invasions by reproductive	χ^2^ (repr.)	*P* (repr.)	Mean proportion colony invading	*F* (colony size)	*P* (colony size)
*Diacamma* #200	3.37	0.000	14	0	**53.3**	**<0.001**	0.141	**25.5**	**<0.001**
*Diacamma rugosum*	3.64	0.033	14	1	**36.8**	**<0.001**	0.052	**86.9**	**<0.001**
*Polyrhachis* #227	3.32	0.040	14	12	0.2	0.642	0.437	1.5	0.221
*Leptogenys* #068	2.81	0.113	14	14	2.5	0.111	0.571	0.3	0.617
*Gnamptogenys menadensis*	2.00	0.261	14	11	1.2	0.268	0.469	0.7	0.416
*Paratrechina* #092	0.79	0.662	14	14	2.5	0.111	0.622	1.3	0.258
*Pheidole* #028	0.73	0.699	14	11	1.2	0.268	0.510	0.1	0.771
*Crematogaster* #085	0.62	0.772	14	14	2.5	0.111	0.714	**6.1**	**0.016**
Control (empty fern)	NA	NA	50	45	NA	NA	0.534	NA	NA

Statistically significant comparisons are presented in bold. Sample sizes are the same for both measures of success, and are presented in the ‘Trials’ column (d.f. for GLMs: treatment = 1, residuals = 63). Test statistics for the effects of species presence compared with empty control ferns are presented as χ^2^ and *F* values for tests against null models.

We first tested whether invasion success differed between occupied and empty ferns. For each resident species in the single species experiments, we ran a generalised linear model (GLM) with a binary predictor (occupied vs. empty control), and either reproductive invasion success or proportion of invading colony successfully entering as the response variable (with binomial and quasi-binomial errors, respectively). For experiments with three resident species, we pooled results from the different combinations of resident species and analysed them with two GLMs (one per response variable) in which the identity of the residents in each trial was defined by six binary factors, one for each of the possible resident species. We simplified the models by backward-stepwise elimination to obtain minimal AIC (or QAIC for the proportion of colony invading). Finally, we explicitly tested for the role of body size rather than species identity. Using the same response variables, we built two GLMs with difference in log_10_ body size between the invader and the resident species as our predictor, using the same error families as above.

### Does size-based competitive assembly drive community structure and is the precise nature of the assembly rule important?

To test whether body-size based competitive assembly could predict the community structure of highly diverse fern-dwelling ant communities, we simulated species assembly under a range of models, and compared the community structure created by these simulations to that observed in wild communities. In particular, we wanted to know whether these rules might comprise a stabilising mechanism *sensu* Chesson (2000), whereby rare species tend to increase in abundance, and common species tend to decrease in abundance (as measured by the diversity of the community relative abundance distribution). We used a null assembly rule, in which species assembly was independent of body size, and a series of alternative rules in which assembly depended on body size.

#### Species assembly rules

Simulated communities were created by drawing individual ant colonies from a species pool (see below for details), assigning them a fern to colonise, and applying the following assembly rules. The probability of successful invasion into a fern was defined as a function of ΔS, the difference in log_10_ body size between the invader and the resident species | *log*_10_(*invader* · *size*) − *log*_10_(*defender* · *size*) |, with the mean probability of invasion across all body size differences being fixed at that observed from the experimental invasions for all rules. We used three functions: (1) A uniform function (*uniform assembly rule*), our null model for these simulations, in which probability of invasion success was uniform and independent of ΔS (Fig. 2a). (2) A step-function (*threshold assembly rule*), in which the probability of successful invasion was zero for all ΔS below a critical value and one for all ΔS larger than this value (Fig. 2b). This is expected if species occupy niches with ‘hard’ edges, following Hutchinson's (1957) hypothesised rectangular niches, and hence strength of competition is a step function in relation to ecological similarity, with species only interacting when they are more similar than some critical value. (3) A logarithmic function (*saturating assembly rule*) characterising the relationship between ΔS and probability of reproductive invasion success in experimental invasions (Fig. 2c). This is expected if competition increases monotonically with ecological similarity, with invasion probability decreasing in the same manner (e.g. Abrams *et al*. 2008). The uniform and threshold assembly rules were parameterised such that the mean probability of invasion success over the total range of body sizes is the same as for the saturating assembly rule. Note that the shapes of these curves are determined solely from experimental data, not from the wild community patterns with which the simulation results are compared.

Incoming species might experience diffuse competition from all residents, with any single resident competing less strongly in a mixture than as sole occupant (Pianka 1974). Alternatively, the main resistance to colonisation might derive from only the most ecologically similar ‘nearest neighbour’ species (Mouillot *et al*. 2007). To determine whether assembly rules based on *nearest neighbour competition* or *diffuse competition* best predicted community patterns, we defined probability of invasion as depending only on the ΔS between the invader and the most similar-sized resident species (nearest neighbour competition), or on the mean of the probabilities predicted by the ΔS between the invader and all resident species (diffuse competition). Hence, we used one null assembly rule, in which assembly was independent of body size difference, and four combinations of rules in which assembly depended on body size difference: threshold/saturating assembly × nearest neighbour/diffuse competition.

#### Simulations

We consider a community of 1000 ferns, with sizes bootstrapped (sampled with replacement) from the size distribution of our 86 ferns. To allow comparison with empirical data, our simulated community included a complete set of 86 ferns matching the original size distribution. We based our source meta-community (which provided the species pool for creating the simulated fern communities) on the observed ant community, maintaining the same species richness as that observed, and the same rank order of abundances (colony presences) in relation to ant body size. Since we wished to determine whether the assembly rules described above could stabilise community structure, and whether or not any resulting stable communities resembled those observed, we varied the relative abundance distribution of the starting meta-community. The observed abundance distributions of ant species across ferns (colony occurrences) did not differ significantly from a log-normal distribution, a property found in many natural communities, so we drew our meta-communities from a log-normal distribution. Variance was drawn from a uniform distribution such that a measure of species diversity, the probability of any two randomly selected colonies being from different species (Simpson's diversity index, 1−D, referred to as ‘diversity’ for brevity), spanned a range bounding the observed community (0.90−0.98, Fig. S1). We used Simpson's index since it is sensitive to changes in abundance distributions (Magurran 2004). The mean of the distribution was set such that the resulting community had the expected number of species occurrences given the numbers and sizes of ferns. Body sizes were assigned to the resulting abundances following their abundance ranking in the observed community.

During each time step of the model, we simulated the recolonisation of a whole community of empty ferns by selecting species at random from the meta-community weighted by their abundance (for the first step, the source meta-community, and for subsequent steps, the community from the previous time step) and assigning them to ferns with probabilities relating to the size of the fern (the number of occupants in the observed community). Probability of successful colonisation depended on the body sizes of existing resident species as described above. This was repeated until the number of species occurrences in the simulated community was the same as that in the source community (i.e. total community size remained the same).

For each assembly rule, we ran 10 000 simulations of 25 assembly cycles (time steps) each. At the end of each assembly cycle, we selected 86 ferns with the same sizes as the empirical set and computed community diversity. Pilot simulations confirmed 25 cycles to be sufficient to stabilise this quantity for all assembly rules of interest (with the exception of the null rule, which is, by definition, stochastic). To avoid gradual reduction of diversity due to local extinction of species, we reintroduced extinct species in the following time step with abundance of 1 (species are unlikely to go extinct in the wider community). Support for the different assembly rules was quantified by Bayes factors estimated using Approximate Bayesian Computation, using a rejection sampling approach where 5% of the best simulations (irrespective of the rule used) were retained (giving an ε ball value, the maximum difference between observed and predicted diversity in the accepted set, of 0.01).

## Results

### Observations of ant co-occurrence patterns in relation to body size

**Figure 1:**
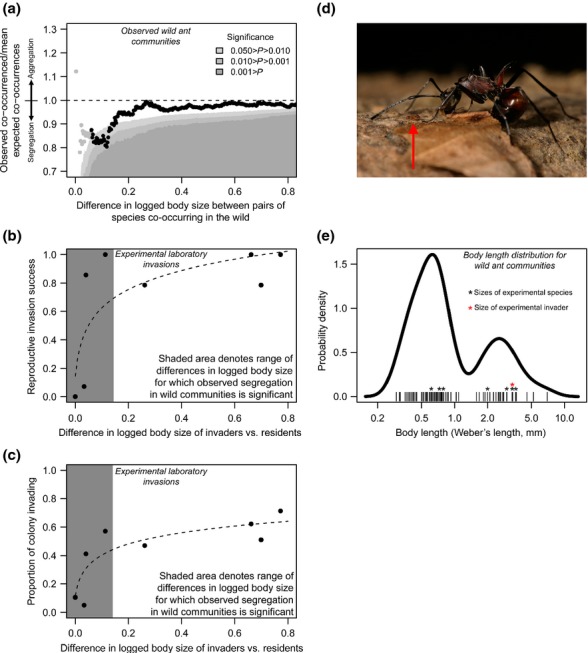
(a) There was less co-occurrence in wild fern-dwelling ant communities than would be expected for similar-sized species. Each point in the plot represents the deviation away from the degree of co-occurrence expected at random (= 1) for all pairs of species with a size ratio more similar than that *x*-axis value. Light grey points indicate the range of size differences for which there was low power due to low numbers of pairs of species (see Material and Methods). In laboratory-based experimental colonisations of ferns containing one resident ant species, only similar sized residents resisted the invader as measured by both (b) probability of reproductive individual invading and (c) proportion of colony invading. Note that figures (a–c) have the same *x*-axis scale. (d) A small *Pheidole* worker (left, red arrow) and a large *Polyrhachis* worker (right). (e) The observed body size distribution for the 71 species of fern-dwelling ants.

The 71 ant species found in the 86 ferns (mean = 2.8 species per fern) showed significant levels of segregation between similar-sized species up to a threshold difference of ∼ 0.13 in log_10_ body size (Fig.1a). This corresponds to a body size ratio of 0.87, that is, there were significantly low levels of co-occurrence between species whose body size differed by < 13%. Species with very small differences in log_10_ body size (< 0.06) were not significantly less likely to co-occur, although for the majority of these comparisons statistical power was low (grey points in Fig.1a). A test for phylogenetic relatedness confirmed that these results are driven by body size, and not taxonomic similarity (Fig. S2). There was no relationship between ant body size and segregation between similar-sized species (Fig. S3).

### Invasion of experimental fern-ant communities

Invading colonies experienced competition in the form of aggressive defence of ferns by resident species (Fig.1, Appendix S3). For experimental ferns with a single resident ant species, the presence of the two species most similar in size to the invader, *Diacamma* #200 and *Diacamma rugosum*, led to a lower probability of successful reproductive invasion and a decrease in the proportion of the invading colony remaining after 24 h compared to empty (control) ferns (Table 1, Appendix S3). In contrast, the presence of *Crematogaster* #085, the species most dissimilar in size to the invader, led to an increase in the proportion of the invading colony remaining after 24 h relative to empty ferns (Table 1). Pooling the results across all resident species, the invasion probability (as estimated by both our measures, Fig.1b and c) decreased with smaller differences in size between the resident and the invader (Reproductive invasion success: χ^2^ = 32.2, d.f. = 1, 6, *P* < 0.001 coefficient for effect of size = 5.04, proportion colony invading: χ^2^ = 78.3, d.f. = 1, 6, *P* < 0.001, coefficient for effect of size = 2.1), with the effect being strongest at differences in log_10_ body size of < 0.1 (approximately the level of similarity that led to low co-occurrence in wild communities).

The interspecific interactions detected in the single species ferns were also found in the fern communities with three resident species: a model including both *D. rugosum* (negative effect on invasion probability) and *Crematogaster* #085 (positive effect) provided the best fit for both measures of invasion success (Table 2). Co-efficient sizes indicated that for *D. rugosum* the effect of the presence of this species was negative, although greater in single species ferns than in three species ferns (Model coefficients: Reproductive invasion success in single species ferns: −4.78, three species ferns = −1.69; Proportion of colony invading in single species ferns: −3.04, three species ferns = −0.72). Thus, the single species results scale well to larger communities, providing a solid foundation for our simulations.

**Table 2:** The identity of the resident ant species affected experimental invasion probability by *Diacamma* #200 for ferns inhabited concurrently by three different ant species (GLMs with quasi-binomial error structure)

		Reproductive invasion success		Proportion colony remaining	
Resident species	Difference in log_10_ body size	*z*	*P*	*T*	*P*
*Diacamma rugosum*	0.033	**−2.055**	**0.040**	**−2.671**	**0.009**
*Polyrhachis* #092	0.040	0.520	0.603	0.849	0.398
*Gnamptogenys menadensis*	0.261	**−**0.864	0.388	**−**1.712	0.091
*Paratrechina* #092	0.662	**−**0.864	0.388	**−**0.848	0.399
*Pheidole* #028	0.699	1.201	0.230	1.705	0.092
*Crematogaster* #085	0.772	**1.426**	**0.153**	**2.405**	**0.019**

Values relating to species remaining in the final minimal AIC/QAIC model are present in bold.

### Simulation of body-size based ant species assembly

**Figure 2:**
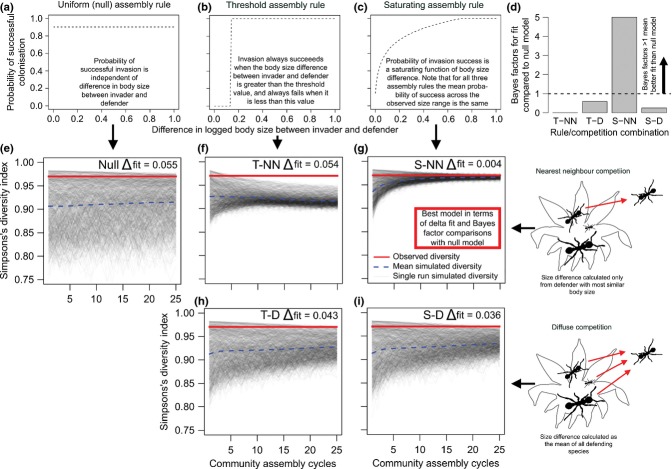
The saturating assembly rule in combination with nearest neighbour competition resulted in communities with species diversity most similar to that of the real communities. Three rules (a–c) relating body size difference to colonisation probability were used to simulate community assembly. Community trajectories in terms of Simpson's diversity are presented over the course of 25 community assembly cycles, with 1000 replicates per rule combination. Panels (f) and (g) show behaviour under the assembly rules assuming that only the most similar sized species interact (nearest neighbour competition) and (h) and (i) all resident species interact (diffuse competition). The results of the null model (e) are the same for nearest neighbour and diffuse competition (by definition) and hence are presented only once. Panel (d) shows Bayes factors for comparisons of fits to the observed diversity of wild communities of the four different size-based assembly models with the null model.

Communities simulated using the null rule, for which assembly was independent of body size, had a wide range of diversities (difference between mean simulated vs. observed diversity, Δfit = 0.055, Fig.2e). Communities simulated using the body-sized based assembly rules (Fig.2f–i) stabilised to a narrower range of diversities over 25 cycles, but varied in their ability to match observed community diversity compared with the null model. Three assembly rule combinations were no more successful than the null model (Threshold-nearest neighbour: Δfit = 0.054, Bayes factor = 0.000; Threshold-diffuse: Δfit = 0.043, Bayes factor = 0.592; Saturating-diffuse: Δfit = 0.036, Bayes factor = 0.254), while the remaining combination, the saturating assembly rule combined with diffuse competition, was markedly better at matching real community diversity than the null model (Δfit = 0.004, Bayes factor = 5.002). Note that the lack of convergence of the null model, resulting in a high variance in community diversity after 25 generations, means that this model is evaluated as performing better than the threshold-nearest neighbour model, which converges more strongly, but at a lower diversity than that observed in the wild community.

## Discussion

We have demonstrated that ant species in wild ferns are segregated with respect to body size. Previous analyses of species co-occurrence in this system using traditional null modelling methods in which co-occurrence is not related to body size fail to detect non-random patterns of community structure (Fayle *et al*. 2013). Furthermore, our novel trait-based analysis of species co-occurrence allowed us to detect the range of body size differences over which species compete, with the observed range agreeing closely with that determined using laboratory-based experiments. Previous analytical methods demonstrated that the spacing of ant species from a range of habitats along an axis of body size is more even than would be expected at random (e.g. Ellwood *et al*. in revision; Sanders *et al*. 2007) but were not able to detect how close in body size a pair of species needs to be before they start to competitively interact. With the increasing availability of trait data for entire animal and plant communities (e.g. http://www.try-db.org/), this method has potential to be extended to assess co-occurrence simultaneously along multiple niche axes. Furthermore, the incorporation of environmental data using environmentally constrained null models of species co-occurrence (Peres-Neto *et al*. 2001) would allow quantification of the relative roles of competition and environmental filtering in multi-dimensional niche-space.

Experimental evidence is vital to support the existence of competitive species assembly, and yet such demonstrations are rare (Gotzenberger *et al*. 2011), and mostly focus on sessile organisms, such as plant (e.g. Fargione *et al*. 2003; Fukami *et al*. 2005) or rocky shore communities (Sousa 1979; Underwood 2000). In this study, we found clear evidence that ant species with similar body sizes competitively excluded each other from ferns, and excluded the alternative explanation of phylogenetic similarity (Fig. S2). Although ant community composition in *Asplenium* ferns is affected by the environment, this effect is weak (Fayle *et al*. 2012), and furthermore, environmental filtering is expected to cause clustering of species with similar traits (Savage & Cavender-Bares 2012; Donoso 2014), the opposite pattern to that observed here. Similar-sized species are expected to be more likely to share resources (Davidson 1985), or natural enemies (Gotelli & Ellison 2002), and therefore to experience more competition. One species, *Crematogaster* #085, the most dissimilar in terms of body size relative to the invader, had a positive effect on invasion success, indicating either a mutualistic (Menzel *et al*. 2008) or facilitatory interaction. We did not include positive interactions between species in our simulations of community assembly. However, despite this, our best fit simulation model still generated realistic ant communities. Although competition between species has long been thought of as the ‘hallmark of ant ecology’ (Hölldobler & Wilson 1990), recent experimental work has been more equivocal in demonstrating the importance of that process (Gibb 2011). Our invasion experiments indicate that, at least in this system, there is competition between ant colonies.

By simulating the formation of communities using size-based rules, we were able to show that competitive species assembly along a single niche axis is sufficient to explain observed relative abundance distributions. Our null assembly rule failed to generate high community diversity, despite the same constraints being present in both sets of simulations (total community size, number of species in species pool, body-size abundance ranking). Although competition is known to be important for a range of taxa (Kunstler *et al*. 2012; Price & Pärtel 2013), including ants (Cole 1983) and species are known to assemble on the basis of traits (Davidson 1985; Kraft *et al*. 2008), including for ants colony size (Palmer 2004), to our knowledge, this ability of competitive trait-mediated interactions to generate realistic community-level patterns has not previously been demonstrated for animals (for plants see Volkov *et al*. 2009). The value of such an approach is that, even if strong competition were demonstrated between species of similar sizes, competition might not be important at the community level (in our study, at the level of the entire fern-dwelling ant community, spread across multiple ferns). This would be the case if, for example, very few pairs of species were sufficiently similar in size to compete or if the species that were similar in size were rare. Furthermore, indirect facilitation via suppression of a shared competitor can be stronger than any direct pairwise competition between species (Levine 1999), but will not be observed during pairwise experimental assays of interaction strengths. Critically, the strength of pairwise competitive interactions need not correlate with the importance of those interactions in structuring communities, since the latter also depends on species abundances, encounter rates, and indirect effects via other species. This observation has implications for models of diversity maintenance; although intraspecific competition is expected to be at least as strong (Hubbell 2001) or stronger (Chesson 2000) than interspecific competition, if species are rare then intraspecific interactions will also be rare, and hence intraspecific competition may be less important than interspecific competition in structuring communities (despite being stronger on a per-capita basis). This is particularly likely to be true in highly diverse tropical communities, where many species are rare (Basset *et al*. 2012).

Using our range of experimentally determined size-based species assembly rules, we were able to demonstrate the sensitivity of community patterns to the scope of competition. Simulation models in which the single most ecologically similar species competed with invaders (nearest neighbour competition, Mouillot *et al*. 2007) resulted in stronger convergence on a final diversity than those which allowed all resident species to compete (diffuse competition, Pianka 1974). However, it was only in combination with the saturating assembly rule that nearest neighbour competition predicted a diversity matching that from the wild community. We suggest that competition between pairs of more abundant species that are of similar size may be important in limiting abundances of these most widespread species, and that under a diffuse competition model the direct competition between these pairs is diluted by the presence of other, less similar-sized species in the ferns.

The relative abundance distribution of the simulated communities was also highly sensitive to the shape of the relationship between body size difference and competitive strength. The saturating assembly rule resulted in communities with more similar diversities to the observed data than the threshold assembly rule across all models, despite the two rules being qualitatively similar (similar-sized species compete more strongly). This indicates the importance of both weak competition between the many species that are moderately ecologically similar, and strong competition between more ecologically similar species. However, this effect was stronger for nearest neighbour competition than for diffuse competition. This interaction between the scope of competition and the body size difference/competitive strength relationship demonstrates that different assembly rule mechanisms can combine in a non-additive manner, necessitating the exploration of specific rules as we have done in this paper. Our results also confirm the theoretical prediction that small changes in competitive strengths between pairs of species can have major impacts on community structure and species coexistence (Zhou & Zhang 2008). Furthermore, being able to predict species interactions on the basis of traits, a method that has long been incorporated into food web models (Williams & Martinez 2000), offers the exciting prospect of generating putative competitive interaction networks without conducting experimental assays between all pairs of species in a community.

Understanding how interactions between individuals governed by simple rules are able to generate patterns in high level community structure is an emerging field in ecological research (Purves *et al*. 2013). Here, we have demonstrated the existence of species assembly rules in tropical epiphyte-dwelling ants through experiments and observations, and revealed how such rules can explain ant community structure. Competitive species assembly occurred, and was sufficient to explain patterns in relative species abundance. We envisage that a broad range of other ecological questions will be amenable to this approach of using of trait-derived rules to simulate biotic communities and then comparing these predictions to independently derived data on community structure.
