# Supplementary material for: Experimentally testing and assessing the predictive power of species assembly rules for tropical canopy ants
Source: Ecol Lett. 2015 Jan 27;18(3):254–62. doi: 10.1111/ele.12403 (PMC4342770; doi:10.1111/ele.12403)
Supplement: Supplementary file 1 [file ele0018-0254-sd1.docx]

**Experimentally testing and assessing the predictive power of species assembly rules for tropical canopy ants**

*Supplementary online material*

Tom M. Fayle

Faculty of Science, University of South Bohemia and Institute of Entomology & Biology Centre of Academy of Sciences Czech Republic, Branišovská 31, 370 05 České Budějovice, Czech Republic; Forest Ecology and Conservation Group, Imperial College London, Silwood Park Campus, Buckhurst Road, Ascot, Berkshire, SL5 7PY; email: [tmfayle@gmail.com](mailto:tmfayle@gmail.com). Corresponding author.

Paul Eggleton

Life Sciences Department, Natural History Museum, Cromwell Road, London SW6 5BD, UK; email: p.eggleton@nhm.ac.uk

Andrea Manica

Evolutionary Ecology Group, Department of Zoology, University of Cambridge, Cambridge, CB2 3EJ, UK; email: am315@cam.ac.uk

Kalsum M. Yusah

Institute for Tropical Biology and Conservation, Universiti Malaysia Sabah, 88999 Kota Kinabalu, Sabah, Malaysia; Forest Ecology and Conservation Group, Imperial College London, Silwood Park Campus, Buckhurst Road, Ascot, Berkshire, SL5 7PY; email: kalsum.myusah@gmail.com

William A. Foster

Insect Ecology Group, University Museum of Zoology Cambridge, Downing Street, Cambridge CB2 3EJ, UK; email: waf1@cam.ac.uk

Appendix 1 Supplementary methods

*Field and laboratory methods*

For censuses of fern-dwelling ants, core fragments were placed in Winkler extractors for three days following [Fayle *et al.* (2012](#_ENREF_12)). To conduct laboratory-based invasion experiments, living ant occupants were extracted. For more abundant species, we excised colony centres from the ferns, while ants from less abundant species were removed individually, along with brood. Colonies were kept in plastic containers, and fed with ad lib dead arthropods and sugar solution. We used different ferns for censuses and experimental work. Ant specimens were identified either to named species or to morphospecies within genera with voucher specimens deposited at the University Museum of Zoology, Cambridge, UK.

*Power testing for co-occurrence patterns in the wild*

We quantified potential decrease in power by taking the mean standardised effect size for all size differences up to the largest size difference at which there were no more significant comparisons, and then testing whether the comparisons with smaller sample sizes would have been able to detect a difference of this magnitude.

*Testing for a relationship between body size and segregation between similar-sized species*

We quantified deviations from expected levels of co-occurrence for all pairs of species that were more similar in size than the size different at which the overall comparison was most significant. These were then averaged for each species, and we then tested whether body size (Weber’s length) predicted these standardised effect sizes using a linear model. In order to test whether any size-based co-occurrence might be explained by phylogenetic relatedness, we also conducted a similar analysis using phylogenetic distances rather than size differences (distances based on the phylogeny of [Brady *et al.* (2006](#_ENREF_3)), species within genera represented as terminal polytomies; Figure S2).

Appendix 2 Function in R for running trait-based co-occurrence analyses.

Appendix 3 Video of an attempted colonisation event of a single species fern containing a colony of *Diacamma* # 200. The attacking individual is denoted by a green paint spot on the pronotum.


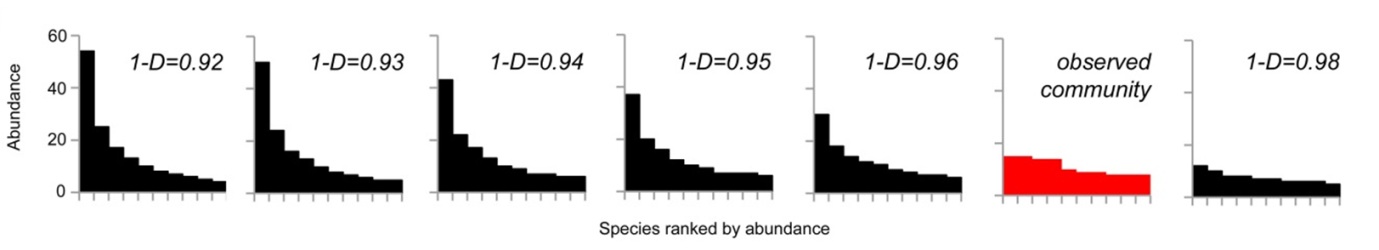


Figure S1 Examples of a range of relative abundance distributions (in black), plus the observed distribution for wild ants (in red 1-D=0.970), ordered by their evenness (probability of drawing two individuals of different species, Simpson’s diversity index, 1-D).

Figure S2 Trait-based co-occurrence analysis relating segregation between species to phylogenetic distance. This analysis was conducted in the same way as that for size differences, but using phylogenetic distance between species in place of size differences. A matrix of relatedness values was constructed based on the phylogeny of Brady *et al.* ([2006](#_ENREF_3)), with species within genera considered as terminal polytomies, since species-level phylogenies are not available for most ant genera, and distances expressed as number of substitutions per site. The result is robust across the whole range of possible assumed intrageneric phylogenetic distances. There was no evidence for species that are more closely related to be segregated in ferns. There is however, a brief run of near significant (P=0.066) data values at around 0.15 substitutions per site.


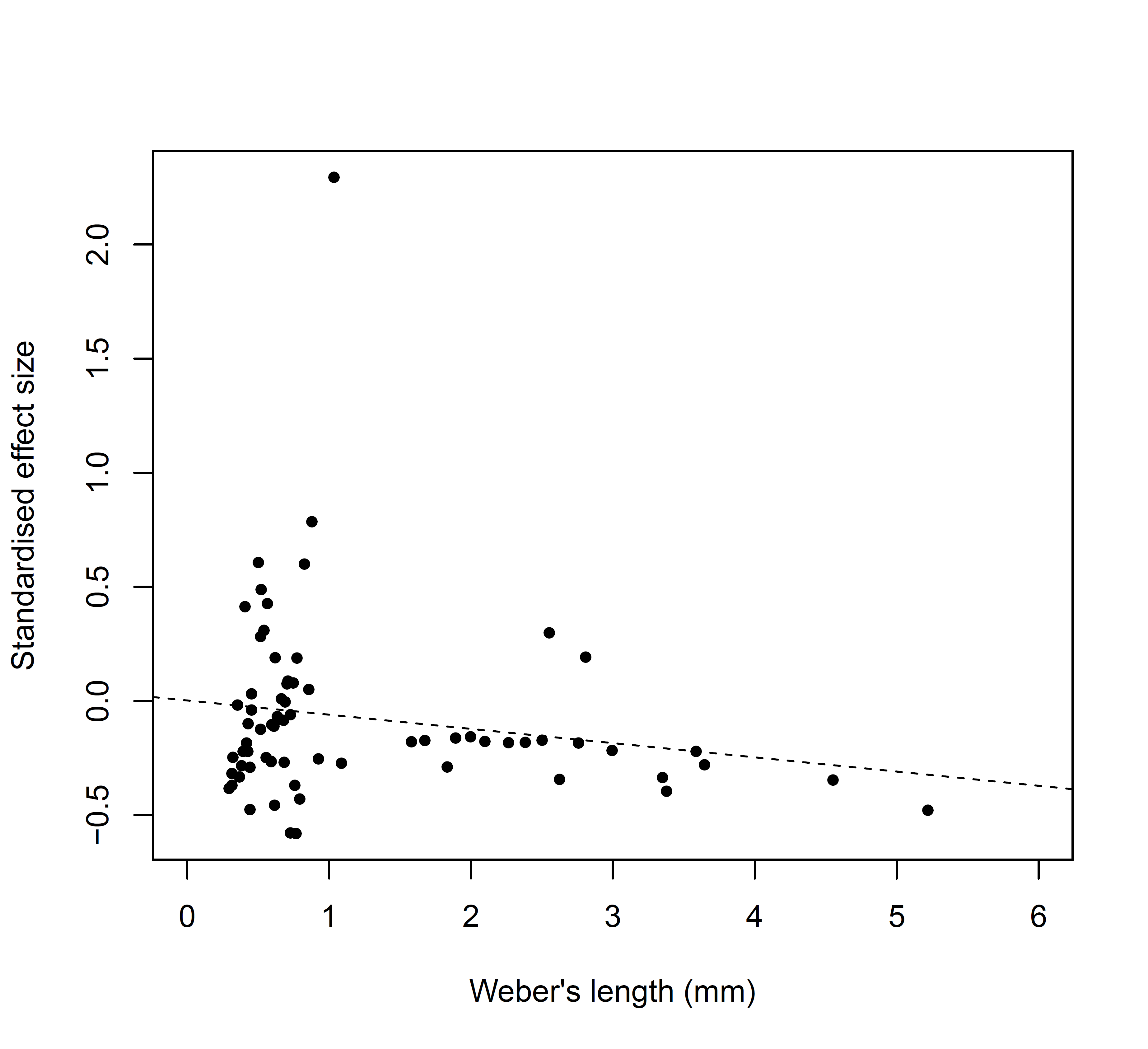


Figure S3. There was no relationship between the body size of each ant species and the mean standardised effect size of that species in terms of its segregation with other similar-sized species (difference in log10 body size <0.115; linear model N=71, t=-1.45, P=0.151). Standardised effect sizes smaller than zero indicate species segregation, while those larger than zero indicate species aggregation. Standardised effect size values are the means for the pairwise interactions between each focal species and all species of similar sizes.
