# Supplementary figures and images for: Experimentally testing and assessing the predictive power of species assembly rules for tropical canopy ants
Source: Ecol Lett. 2015 Jan 27;18(3):254–62. doi: 10.1111/ele.12403 (PMC4342770; doi:10.1111/ele.12403)

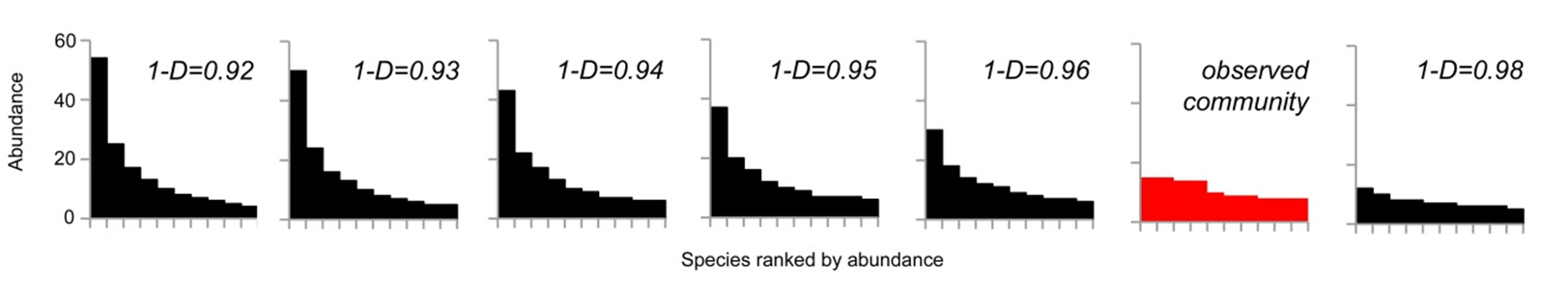

Supplement: Supplementary file 4 [file ele0018-0254-sd4.jpg]

Observed co-occurrences/mean expected co-occurrences

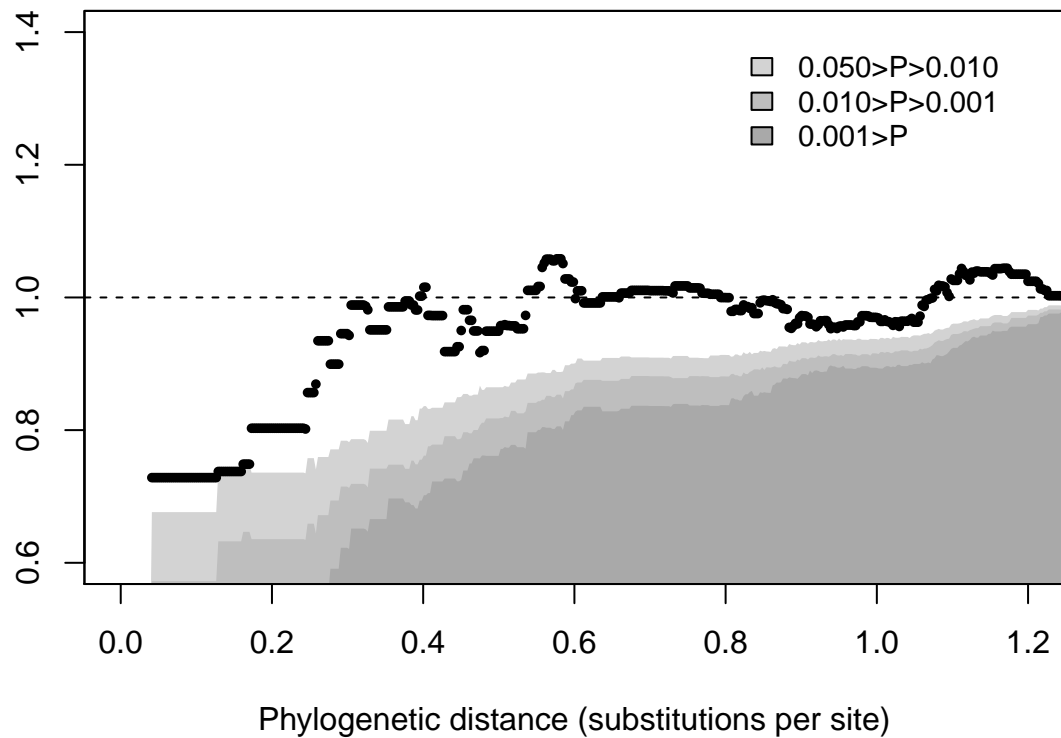

Supplement: Supplementary file 5 [file ele0018-0254-sd5.pdf]

Standardised effect size

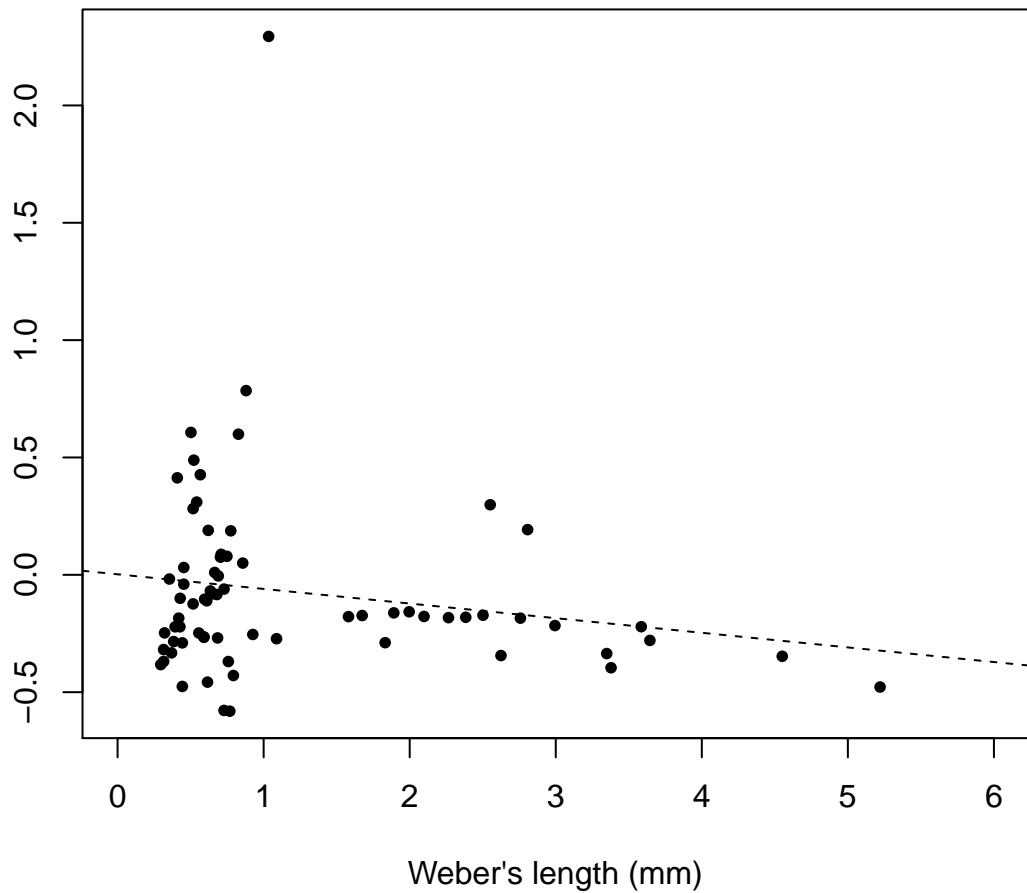

Supplement: Supplementary file 6 [file ele0018-0254-sd6.pdf]
